# Autonomous Navigation by Mobile Robot with Sensor Fusion Based on Deep Reinforcement Learning

**DOI:** 10.3390/s24123895

**Published:** 2024-06-16

**Authors:** Yang Ou, Yiyi Cai, Youming Sun, Tuanfa Qin

**Affiliations:** 1 School of Computer and Electronic Information, Guangxi University, Nanning 530004, China; ouyang@st.gxu.edu.cn (Y.O.); caiyiyi@gxu.edu.cn (Y.C.); ymsun@gxu.edu.cn (Y.S.); 2 The Guangxi Key Laboratory of Multimedia Communications and Network Technology, Guangxi University, Nanning 530004, China; 3 School of Electronic and Information Engineering, South China University of Technology, Guangzhou 510641, China

**Keywords:** sensor data fusion, deep reinforcement learning, autonomous navigation, mobile robots

## Abstract

In the domain of mobile robot navigation, conventional path-planning algorithms typically rely on predefined rules and prior map information, which exhibit significant limitations when confronting unknown, intricate environments. With the rapid evolution of artificial intelligence technology, deep reinforcement learning (DRL) algorithms have demonstrated considerable effectiveness across various application scenarios. In this investigation, we introduce a self-exploration and navigation approach based on a deep reinforcement learning framework, aimed at resolving the navigation challenges of mobile robots in unfamiliar environments. Firstly, we fuse data from the robot’s onboard lidar sensors and camera and integrate odometer readings with target coordinates to establish the instantaneous state of the decision environment. Subsequently, a deep neural network processes these composite inputs to generate motion control strategies, which are then integrated into the local planning component of the robot’s navigation stack. Finally, we employ an innovative heuristic function capable of synthesizing map information and global objectives to select the optimal local navigation points, thereby guiding the robot progressively toward its global target point. In practical experiments, our methodology demonstrates superior performance compared to similar navigation methods in complex, unknown environments devoid of predefined map information.

## 1. Introduction

In the evolution of Simultaneous Localization and Mapping (SLAM), high-precision localization and robust mapping have been achieved. SLAM systems rely on operators controlling measurement devices to collect positional data and environmental features, thus constructing maps [1]. In unknown environments, humans can expedite the arrival of devices at target points by relying on their own experience and understanding of the environment to plan paths. However, this method is limited by factors such as labor costs, potential hazards, and physical constraints. Therefore, automated exploration and navigation path-planning techniques have become a research hotspot. Current research focuses on integrating autonomous navigation with mapping, where mobile robots use map information to autonomously plan a path to the target point via a planner [2]. Algorithms such as A*, Dijkstra [3], RRT, and RRT* [4] utilize existing map information to plan optimal or approximately optimal paths to the destination [5]. However, these methods become impractical in unknown environments. With the application of deep reinforcement learning in robotics, its precise decision-making ability makes it a viable option for autonomous navigation. By employing deep reinforcement learning algorithms to simulate human characteristics and intelligence [6], robots are endowed with the ability to learn action policies based on the current environmental state, enabling effective navigation even in unknown environments after training in diverse environments [7]. This makes deep reinforcement learning particularly suitable for the autonomous navigation tasks of mobile robots. However, popular deep learning algorithms such as DDPG [8], TD3 [9,10], and SAC [11] have limited input state dimensions, and action policies are only relevant to current environmental information, which may lead to policy entrapment in local optima, action loops, or stagnation [12]. Additionally, when the target point is beyond the training range, the robot may fail to navigate correctly. In such cases, intermediate nodes need to be selected between the mobile robot and the target point, gradually approaching the target by continuously selecting intermediate nodes. Heuristic functions play a crucial role in this process, synthesizing various information in the robot’s surrounding environment to score different intermediate nodes and select the optimal navigation node [13].

Deep reinforcement learning requires real-time state information and environmental perception data from the robot during training, which is primarily sourced from the various sensors mounted on the robot. Lidar and camera, serving as the primary perception devices for the robot, are utilized to detect obstacles within the environment and capture surrounding environmental information. Meanwhile, the odometer provides the robot’s positioning information, relative distance, and azimuth angle to the target point. Lidar can be categorized into 2D and 3D types based on its functionality, exhibiting both high precision in object recognition and insensitivity to changes in lighting conditions [14]. Two-dimensional lidar, limited by its scanning plane, cannot detect obstacles above or below its scanning plane; in contrast, 3D lidar can identify obstacles in multiple dimensions but comes with a higher cost. Although cameras have lower costs and can provide rich visual information, their performance is significantly influenced by lighting conditions, making it challenging to obtain clear images in strong or dim light environments [15]. By fusing sensor data from lidar and camera, complementary advantages can be achieved, overcoming their respective limitations and optimizing the performance of the perception system [16].

Therefore, we propose an autonomous navigation system that does not rely on prior information for guiding mobile robots to target locations. This system extracts all potential navigation nodes around the robot and evaluates them to select the optimal navigation node. Subsequently, utilizing the Soft Actor–Critic (SAC) network from deep reinforcement learning to process sensor input data generates motion strategies to reach the selected navigation points and gradually guides the robot to the global target point. This system does not require pre-mapping in unknown environments, effectively reducing the risk of falling into local optima. The main contributions of this work are summarized as follows:

- Designed and implemented an algorithm based on the Soft Actor–Critic (SAC) network architecture, providing continuous action outputs for a four-wheeled mobile robot to navigate toward local goals.
- Proposed a Candidate Point-Target Distance (CPTD) method, an improved heuristic algorithm that integrates a heuristic evaluation function into the navigation system of a four-wheeled mobile robot. This function is used to assess navigation path points and guide the robot toward the global target point.
- Introduced a Bird’s-Eye View (BEV) mapping and data-level fusion approach, integrating a 2D lidar sensor with a camera to identify obstacles at different heights in the environment, thereby enhancing the robot’s environmental perception capabilities.

## 2. Related Work

With the application and proliferation of deep learning algorithms in the robotics field, researchers have begun to explore the use of deep learning technology to address the exploration and navigation challenges of robots. Deep learning technology has shown significant effectiveness in solving robotic motion decision-making problems. In [17], Kontoudis et al. proposed an online motion planning algorithm that combines RRT and Q-learning, optimizing strategies and costs for continuous-time linear systems via model-free Q-functions and integral reinforcement learning. Terminal state evaluation and local replanning were also introduced to enhance real-time performance and adaptability to obstacles. Marchesini et al. [18] proposed a method that combines DDQN with parallel asynchronous training and multi-batch prioritized experience replay, which demonstrated potential as a viable alternative for mapless navigation by reducing training time while maintaining success rates. In [19], Dong et al. proposed an improved DDPG algorithm that dynamically adjusts the exploration factor using limited prior knowledge and an ϵ -greedy algorithm, accelerating the training process of deep reinforcement learning and reducing the number of trial-and-error attempts. Li et al. [20] proposed an improved TD3 algorithm, incorporating prioritized experience replay and designing a dynamic delayed update strategy, which reduced the impact of value estimation errors, improved the success rate of path planning, and reduced training time.

The aforementioned methods are based on single sensors; while they can recognize the environment and navigate correctly, they pose risks when encountering specialized obstacles. In [21,22], a method was proposed to combine ultrasonic sensors and camera, using occupancy grid algorithms to merge camera data and ultrasonic measurements. This enabled the robot to efficiently detect obstacles and accurately reach its target. However, the wide beam angle of ultrasonic sensors can result in erroneous identification of protruding objects on walls when the robot is navigating close to them. Zhang et al. in [23] proposed fusing lidar and RGB-D camera to enhance the accuracy of 2D grid maps, followed by employing an improved A* algorithm for mapping and navigation tasks. In [24], Theodoridou et al. proposed integrating RGB-D and lidar to achieve 3D human pose estimation and tracking, allowing the robot to navigate correctly within an environment without invading personal space. In [25], a method combining lidar and RGB-D camera data was proposed, utilizing GPU for parallel computation to implement an asynchronous update algorithm, thereby enhancing the efficiency of obstacle recognition and data processing speed. However, despite the advantages of this algorithm in efficiency and learning performance, there is also a risk of overfitting, especially when training data are limited or the environment is highly dynamic.

In light of this, deep learning technologies exhibit outstanding performance in handling modular tasks, but they have certain limitations in implementing global end-to-end solutions. Typically, multiple technical challenges are encountered during the transition from simulated environments to real-world scenarios. In response to goal-oriented exploration issues, this study proposes an efficient learning strategy that integrates depth camera information transformation with lidar data fusion and combines it with a global navigation strategy. The target navigation system aims to autonomously navigate to a predetermined global target location in unknown environments and without preset detailed paths, while also possessing the ability to actively recognize and avoid obstacles during the journey. Via this strategic integration, the system can more efficiently utilize the precise distance measurement capabilities of lidar to achieve comprehensive environmental perception and accurate navigation.

## 3. Heuristic SAC Autonomous Navigation System

**Figure 1:**
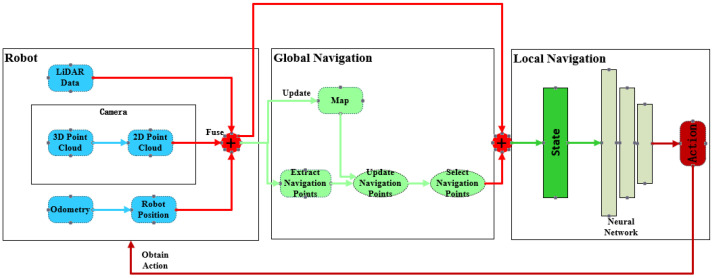
Navigation system architecture. On the left is the configuration of the robot vehicle. In the middle is the process of global navigation point generation. On the right is the process of calculating local navigation actions.

To endow mobile robots with the capability of autonomous exploration and navigation in their environment, the navigation system architecture proposed in this study consists of two parts: local navigation and global navigation. The global navigation module, based on a heuristic function, is used to score candidate navigation points extracted from the environment and select the optimal navigation node from them. Local navigation, on the other hand, is based on the SAC algorithm of deep reinforcement learning, which inputs the selected optimal navigation points in polar coordinate form into a neural network. This neural network integrates the robot’s current position, orientation, and sensor data fusion results to calculate the robot’s immediate action decisions, thereby guiding the robot smoothly to the designated navigation node. The system architecture of this autonomous navigation system is illustrated in Figure 1.

### 3.1. Local Navigation

From Figure 1, it can be observed that we divided the navigation system into three main components. In the local navigation segment, we employed the SAC algorithm from deep reinforcement learning to obtain motion strategies. By independently training the deep reinforcement learning model in simulated environments, we are able to utilize the trained policies to achieve local navigation for the robot.

The SAC algorithm is a deep reinforcement learning technique based on the maximum entropy principle, following an asynchronous policy learning paradigm. This algorithm leverages behavior policy-generated datasets to optimize the target policy, thereby improving sample efficiency, accelerating learning convergence, and reducing training time [26]. Utilizing an Actor–Critic framework, SAC is well suited for handling complex environments with continuous state and action spaces. Unlike traditional deterministic policies, SAC employs a stochastic policy, guiding action selection through probability distributions. This enhances exploration capabilities, helps avoid local optima, and improves global search performance [27]. The strength of the SAC algorithm lies in its explicit optimization of policy entropy, which encourages broader exploration, enhancing adaptability and robustness [28]. The introduction of entropy balances exploration and exploitation, ensuring comprehensive exploration of the State–Action space and boosting learning efficiency. Thus, the SAC objective function, as shown in Equation (1), aims to find a policy that maximizes the expected cumulative reward while also maximizing policy entropy, thereby encouraging the agent to explore a broader State–Action space.

(1) $$\pi^{*}=arg\underset{\pi }{max}\left(\sum_{s,a}\underset{\mathrm{reward}}{\underbrace{R(s,a)}}+\underset{\mathrm{entorpy}}{\underbrace{\alpha H\left(\pi_{s}\right)}}\right)$$

where π is the policy function, which represents the probability distribution of selecting action *a* given state *s*. R(s,a) is the weighted value function of State–Action pairs. α is a regularization coefficient used to adjust the balance between rewards and entropy. $H\left(\pi_{s}\right)$ is the entropy of policy π , calculated as follows:

(2) $$H\left(\pi_{s}\right)=-\log\left(\pi (a|s)\right)$$

It measures the uncertainty of the policy; a high entropy value implies a higher exploratory nature of the policy, which means there is greater randomness in the actions taken by the robot.

The near-field environment of the robot is described by the fusion of data from the lidar sensor and the camera within a 180° range in front. This fused information is divided into 40 groups and combined with the robot’s position and orientation relative to the navigation point, as well as the robot’s execution steps, to form the input state of the Actor network. The Actor network consists of four fully connected layers (FC), with ReLU (Rectified Linear Unit) activation functions introduced after the first two fully connected layers to ensure positive output values. The last two fully connected layers serve as the output layer, providing the logarithmic values representing the mean and standard deviation of the actions. By sampling the mean and standard deviation of the output, the robot’s linear and angular velocities can be obtained. Finally, the obtained action vector is normalized using the tanh activation function to ensure its values are within the range (−1, 1). Since only forward sensor data of the robot is used, the policy does not consider backward actions, thus requiring adjustment of the linear velocity value to be positive, as shown in the following formula:

(3) $$a=\left[\frac{a_{1}+1}{2},a_{2}\right]$$

where $a_{1}$ is the linear velocity of the robot, and $a_{2}$ is the angular velocity.

**Figure 2:**
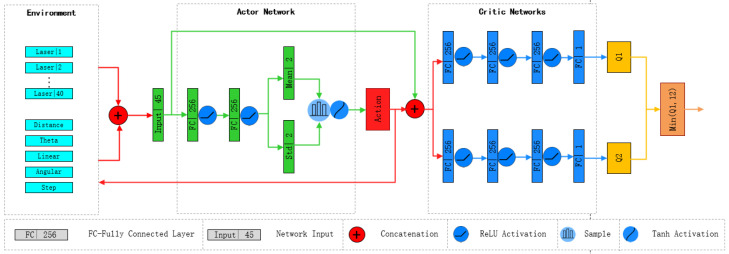
SAC network block diagram. The block diagram includes the robot’s surrounding environment, Actor network, and Critic network.

The Critic network consists of two structurally identical independent Q networks ( $Q_{1}$ and $Q_{2}$ ), both responsible for evaluating the value of specific State–Action pairs. Each Q network comprises four fully connected layers, incorporating ReLU activation functions within the network to introduce nonlinearity. The Critic network takes a pair of states (*s*) and actions (*a*) as input, processing them through three fully connected layers, each layer introducing a ReLU activation function. The input states (*s*) and actions (*a*) are directly fed into the network, and the output of the network is an estimate of the Q value for the corresponding State–Action pair. Finally, the output of the Critic network is derived from the minimum Q value output of the two Q networks, serving as the ultimate evaluation result. The specific network architecture is illustrated in Figure 2.

In the field of reinforcement learning, the reward function plays a central role, serving as a crucial factor driving the optimization of agent behavior strategies [29]. The reward function specifies the immediate reward obtained by the agent when taking a certain action in a specific state, forming the fundamental basis for the agent to evaluate its behavioral effectiveness and make decisions. The design of the reward function is directly related to the learning efficiency of the agent and its ultimate learning performance. Therefore, a properly designed reward function can effectively enhance the learning speed of the agent and optimize its performance.

The reward function proposed in this article is as follows:

(4) $$R\left(s_{t},a_{t}\right)=\left\{\begin{array}{cc} r_{g} & \mathrm{if}\;\mathrm{target}\\ r_{n} & \mathrm{if}\;\mathrm{collision}\\ r_{a} & \mathrm{if}\;\mathrm{not}\;\mathrm{collision}\;\&\;\mathrm{not}\;\mathrm{target}\;\&\;t=500\\ a_{1}-\left|a_{2}\right| & \mathrm{otherwise} \end{array}\right.$$

The immediate reward *r* obtained by the State–Action pair $\left(s_{t},\;a_{t}\right)$ at timestep *t* depends on four conditions. If the robot reaches the target position at timestep *t*, it is awarded a positive reward $r_{g}$ to incentivize successful task completion; if the robot experiences a collision, it is given a negative reward $r_{n}$ as a penalty for engaging in hazardous actions. If, after 500 timesteps, the robot has neither encountered a collision nor achieved the goal, it is similarly granted a negative reward $r_{a}$ to encourage more efficient environmental exploration. If none of the above conditions are met, the immediate reward will be determined based on the difference between the absolute value of the robot’s current linear velocity $a_{1}$ and angular velocity $a_{2}$ to reflect the robot’s motion efficiency and control smoothness. With such a reward mechanism design, the reinforcement learning algorithm can guide the robot to learn strategies that avoid collisions and reach the goal quickly and effectively while exploring the environment.

The network, once trained, achieves a generalized local navigation policy that efficiently reaches local goals while ensuring the safety and feasibility of the path by avoiding obstacles directly detected through lidar and camera inputs. In this strategy, the network utilizes the fused data from lidar and camera to construct a local representation of the environment and, based on this, optimizes path planning to accomplish navigation tasks within complex settings.

### 3.2. Global Navigation

Due to the absence of prior map information, it is not possible to directly obtain the robot’s path to the global target point. Therefore, the robot must first extract available navigation points from the environment to guide its journey to the global target point. Secondly, a heuristic function is used to select the navigation point with the lowest score as the intermediate point towards the global target point. Finally, to ensure its smooth arrival at the designated global target position, an exploration mechanism should also be embedded to explore unknown areas when encountering blocked paths, thereby finding new paths leading to the global target point. To extract navigation points, this study adopts the following three methods:

(1) When the distance between obstacles measured by two consecutive lidar readings exceeds the width of the robot, and the continuous lidar readings between these two readings are all greater than the values of these two readings, a navigation point is generated at the midpoint between these two lidar readings.
(2) In environments with fewer obstacles, if continuous lidar readings indicate a larger free space, navigation points can be generated at a certain interval.
(3) Considering that the detection range of sensors is limited, when the lidar detection values return a non-numeric type (NaN), it indicates that the obstacles have exceeded the sensor’s detection threshold, thus indicating the presence of a relatively spacious area. In this case, if there are non-numeric detection values between two lidar readings, a navigation point is generated at the midpoint between these two readings.

**Figure 3:**
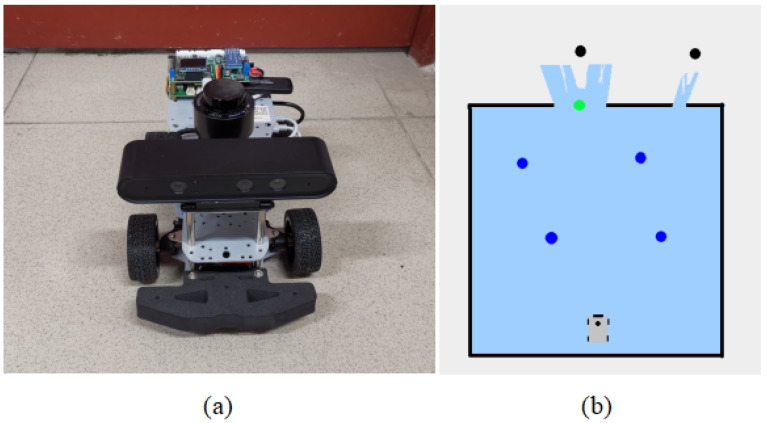
Subfigure (**a**) is the robotic car used in this study. Subfigure (**b**) is an example of environment navigation point extraction. The green dot is the navigation point extracted by method 1. The blue dot is the navigation point extracted by method 2. The black dots are the navigation points extracted by method 3.

Via these extraction strategies, navigation points can be effectively extracted from the environment, providing support for the robot’s autonomous navigation and exploration. Figure 3b illustrates the process of directly extracting navigation points from sensor data using the three aforementioned strategies, which do not rely on any prior environmental information. To optimize the candidate navigation point set, the system automatically excludes navigation points that are too close to obstacles. Additionally, to avoid redundancy, the system does not generate additional navigation points in the vicinity of existing navigation points. This improves the quality of navigation points, ensuring the efficiency and safety of robot path planning.

In the absence of sufficient environmental information, heuristic methods can effectively approximate the optimal solution to a problem. Based on this premise, the CPTD algorithm proposed in our study improves the scoring mechanism in [13], which evaluates all candidate navigation points through a heuristic function to select an optimal navigation point. The robot progressively moves towards each selected navigation point until reaching the global target point. At each step, the robot obtains its own position coordinates, coordinates of each candidate navigation point, coordinates of the global target point, and map data, enabling the calculation of the distances from the robot to each candidate navigation point and the global target point. Subsequently, the score of each candidate navigation point is computed based on the integrated map data. To enable the robot to reach the global target more quickly, we incorporate the previously selected candidate point as an evaluation factor in the function, ensuring that the robot will not choose a candidate point that is farther from the target under normal conditions. Given this information, the score, h of the *i*-th candidate navigation point, $c_{i}$ , is determined as follows:

(5) $$h_{i}=S_{i,t}+\frac{1}{2}[D\left(c_{i},u\right)+D\left(m_{t},u\right)]+M_{i,t}$$

where $S_{i,t}$ is the distance score obtained based on the current position of the robot and the candidate navigation point, calculated as shown in Equation (6). $D\left(c_{i},u\right)$ is the Euclidean distance from the candidate navigation point to the global target point, calculated as shown in Equation (7). $D\left(m_{t},u\right)$ is the Euclidean distance from the previously selected candidate point to the global target point, calculated as shown in Equation (8). $M_{i,t}$ is the map information surrounding the candidate navigation point.

(6) $$S_{i,t}=\tanh\left(\frac{e^{{\left(\frac{D(m_{t},c_{i})}{d_{1}}\right)}^{2}}}{e^{{\left(\frac{d_{2}}{d_{1}}\right)}^{2}}}\right)d_{2}$$

where $D(m_{t},\;c_{i})$ is the Euclidean distance between the robot’s current position and the candidate navigation point. $d_{1}$ and $d_{2}$ are the internal and external distance limits of the heuristic discount, which are set based on the environment trained by the SAC network. They serve to suppress the increase of the heuristic function, causing it to stabilize near the maximum and minimum scores. *e* is Euler’s number, and tanh is the hyperbolic tangent function.

(7) $$D\left(c,u\right)=\sqrt{{\left(c_{x}-u_{x}\right)}^{2}+{\left(c_{y}-u_{y}\right)}^{2}}$$

where *x* and *y* are the x coordinate and y coordinate of the point, respectively. $\left(u_{x},u_{y}\right)$ is the end point coordinates.

(8) $$D\left(m_{t},\;u\right)=\sqrt{{\left(m_{x}-u_{x}\right)}^{2}+{\left(m_{y}-u_{y}\right)}^{2}}$$

where $\left(m_{x},\;m_{y}\right)$ are the coordinates of the robot at step *t*.

Since there is no prior mapping, the robot can only acquire environmental information in real-time through its sensors. With each action, the robot’s map data are continuously refined in real-time. When a candidate navigation point is located within a known environment, the amount of information gained upon reaching that point is significantly less than that of a point situated in an unknown environment. Therefore, we prefer to guide the robot towards navigation points in unknown environments, which aids in discovering potential paths to the global target. Consequently, it is necessary to heuristically score each candidate navigation point based on the map data obtained. The status of every pixel in the map is identified, with unknown pixels marked as $p_{u}$ , obstacles as $p_{o}$ , and known pixels with no obstacles marked as $p_{f}$ . To better reflect the score differences between known environments, unknown environments, and obstacles, we set the divisor to *k* to calculate the map information around the candidate point. The information score, M , for the environment within a [*k* × *k*] window around the candidate navigation point, ${\;c}_{i\;}$ , is calculated as follows:

(9) $$M_{i,t}=\frac{\sum_{j=-\frac{k}{2}}^{\frac{k}{2}}\sum_{n=-\frac{k}{2}}^{\frac{k}{2}}s_{\left(x+j)(y+n\right)}}{k}$$

where *x* and *y* are the coordinates of the candidate navigation point, and $s_{\left(x+j)(y+n\right)}$ is the pixel identifier around the candidate navigation point.

To enhance the distinction between unknown areas, known areas, and obstacles, an exponential function is employed to measure and calculate the final evaluation score for each candidate point:

(10) $$e^{M_{i,t}}$$

Taking into account the scoring methods above, the final heuristic evaluation function for candidate navigation points is as follows:

(11) $$h\left(c_{i}\right)=\tanh\left(\frac{e^{{\left(\frac{D\left(m_{t},c_{i}\right)}{d_{1}}\right)}^{2}}}{e^{{\left(\frac{d_{2}}{d_{1}}\right)}^{2}}}\right)d_{2}+\frac{1}{2}\left[D\left(c_{i},\;u\right)+D\left(m_{t},\;u\right)\right]+e^{M_{i,t}}$$

**Figure 4:**
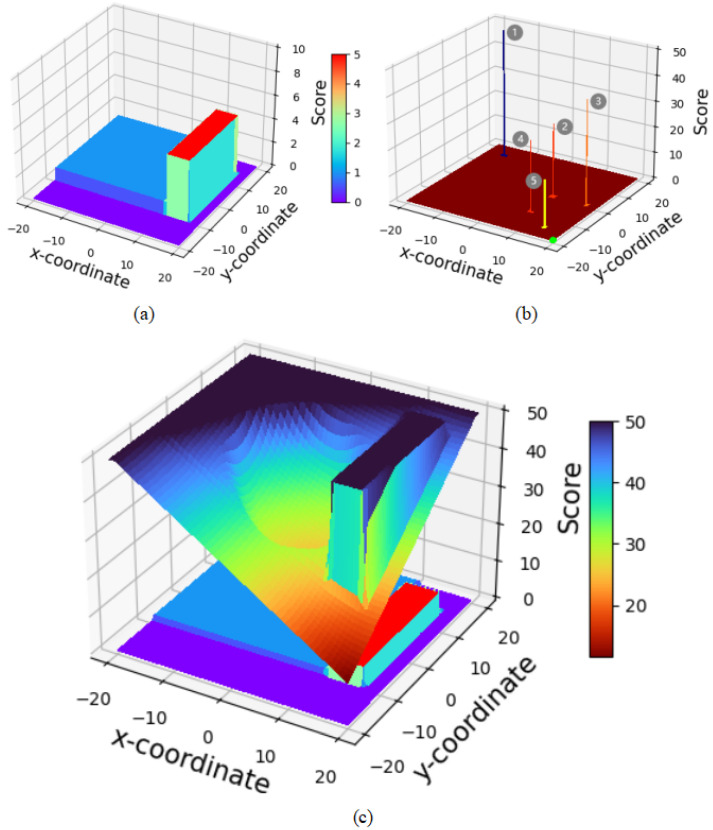
Scoring example of the CPTD algorithm. The x and y coordinates are represented in meters. In (**a**), the z-coordinate represents the identification score of the pixels. The z-coordinate in (**b**,**c**) represents the score given by the heuristic function. (**a**) visualizes the environmental information. (**b**) displays the scores for four points, with the green point being the target point and the robot positioned at the origin (0,0). (**c**) shows the overall scores.

According to the above evaluation function, even if a candidate navigation point is relatively distant from the robot, if it is closer to the global goal or there is still a significant distance between the robot and the global goal, its priority will be higher than that of candidate points near the robot. Additionally, the evaluation mechanism should favor selecting candidate points in unexplored areas. Therefore, the heuristic scoring function is used to filter out the navigation points with the lowest scores and determine them as the highest priority navigation point. Examples of obstacle and scoring representations for the heuristic function are shown in Figure 4.

As shown in Figure 4, subplot (a) reveals the distribution of scores in the environmental map under the given pixel identification conditions, where $p_{u}=0$ , $p_{f}=1$ , and $p_{o}=5$ . Subplot (b) then presents the scoring of candidate navigation points 1 (−15, 15), point 2 (8, 0), point 3 (17, 0), point 4 (8, −10), and point 5 (15, −15) within the environment depicted in subplots (a), with the specific scores detailed in Table 1. Analysis from subplots (b) and (c) allows for the observation that the closer a candidate navigation point is to the target point, the lower the score it receives. Additionally, if a candidate navigation point is near an unexplored area, it will receive a lower score compared to points that are further away from unexplored areas.

### 3.3. Sensor Data Fusion

Sensor data fusion can enhance the reliability and robustness of data, strengthen perceptual capabilities, improve data accuracy and spatial efficiency, and reduce costs and resource consumption [30]. The price of a 3D lidar is significantly higher than the combined cost of a 2D lidar and a camera, yet the latter combination can also capture comprehensive perceptual information about the environment ahead. Therefore, we opted to integrate a 2D lidar with a camera as the perception apparatus for the robot.

To achieve data-level fusion between 2D lidar sensors and cameras, the first step is to perform a Bird’s-Eye View (BEV) projection [31] on the 3D point cloud captured by the camera, transforming complex three-dimensional scenes into a two-dimensional representation. In the Robot Operating System (ROS), there are specific packages designed to convert 3D point clouds into 2D point clouds. This process involves removing point cloud data that exceeds a predefined robot height threshold, correcting ground inclination, and ultimately compressing the 3D point cloud into a two-dimensional plane through dimension reduction to generate the 2D point cloud data corresponding to the camera. Sensor data-level fusion refers to the integration of raw observational data captured by the sensors. During the execution of sensor data fusion, it is essential to spatially calibrate the data collected by the lidar and the camera to ensure accurate correspondence between the data at the same angles. Given that the camera’s field of view is narrower than the lidar’s scanning angle, the formula for matching the starting position of the fused data with the camera is as follows:

(12) $$d_{i}=\frac{l_{s}-l_{c}}{2}$$

where $l_{s}$ is the length of the 2D point cloud data generated by the lidar, and $l_{c}$ is the length of the 2D point cloud data after conversion by the camera.

**Figure 5:**
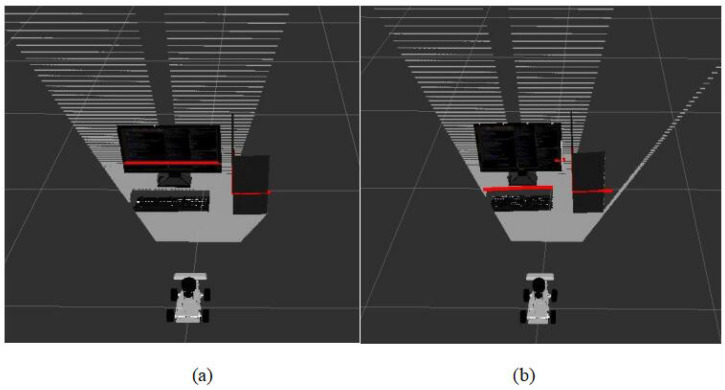
Sensor data fusion. The red lines in the figure represent the range of objects scanned by the sensors. Subfigure (**a**) shows the range of objects scanned only by the lidar sensor, while subfigure (**b**) illustrates the range of objects scanned after fusion of the lidar sensor and camera.

Because lidar is less affected by environmental factors, if it detects an obstacle ahead, the robot must avoid this obstacle. However, if there is an object below the lidar’s scanning range in front of the obstacle, the camera is required to identify this object. The camera serves as an auxiliary sensor, specifically for detecting objects within the lidar’s blind spots. Therefore, if the 2D point cloud data converted by the camera is smaller than the point cloud data from the lidar at the same angle, the camera data are used to replace the lidar data. The effect of sensor fusion is illustrated in Figure 5. Since the fused 2D point cloud consists of 720 data, directly using them as input for the SAC network would significantly increase computational complexity, thereby increasing training time and reducing convergence speed. To ensure efficient training, these point cloud data need to be preprocessed. We divided the fused 2D point cloud data into 40 subsets and extracted the minimum value from each subset, constructing 40 sets of sensor data. These 40 sets of data represent the features of objects closest to the robot in the environment and are used as input to the SAC network. This approach not only prevents collisions between the robot and objects but also enhances data processing efficiency. Algorithm 1 presents the pseudocode for sensor data fusion.
**Algorithm 1** Sensor data fusion
  1:Receive lidar point cloud data → laser  2:Receive camera point cloud data → camera  3:laser → fusion  4:int(length(laser.data) − length(camera.data))/2 → index_start  5:**for** index_camera from 0 to length(camera.data) **do**  6:    index_start + index_camera → index_laser  7:    laser[index_laser] → laser_data  8:    camera[index_camera] → camera_data  9:    **if** camera_data < laser_data **then**10:        camera → fusion11:    **end if**12:    **if** fusion is *∞* **then**13:          10 → fusion14:    **end if**15:    **if** fusion is NAN **then**16:          0 → fusion17:    **end if**18:**end for**


## 4. Experiments and Results

### 4.1. Device and Parameter Settings

The experiment was conducted using a computer equipped with an NVIDIA RTX 3060 graphics card (Santa Clara, CA, USA), 64 GB of RAM, and Intel Core i5-12490F CPU (Santa Clara, CA, USA) to execute deep reinforcement learning algorithms for learning local navigation strategies. The training of the SAC network was conducted in an environment built within the Gazebo simulation software (Gazebo 9). During the training process, each episode ended when the robot reached the target, collided, or executed 500 steps. The maximum linear velocity of the robot was set to 1 m/s, and the maximum angular velocity was set to 1 rad/s. For candidate navigation points, the internal and external discount limits for the heuristic function were set to $d_{1}=5$ m and $d_{2}=10$ m, respectively. The calculation of environmental information for candidate points was set to internal. In the real-world experiment, the platform used is an Ackermann mobile robot, as shown in Figure 3a. It was equipped with a microcomputer core based on Jetson Orin Nano 4G, running on the Ubuntu 20.04 operating system, for executing autonomous navigation programs. It was also equipped with a Leishen N10P lidar sensor (Shenzhen, China) and an Astra_s stereo depth camera (Shenzhen, China) for object recognition in the environment. Additionally, a laptop computer running on the Ubuntu 18.04 system, equipped with an NVIDIA RTX 3060 graphics card, 16 GB of RAM, and AMD Ryzen7 5800H CPU (Sunnyvale, CA, USA), was used for remote monitoring and controlling of the robot car.

### 4.2. Network Training

**Figure 6:**
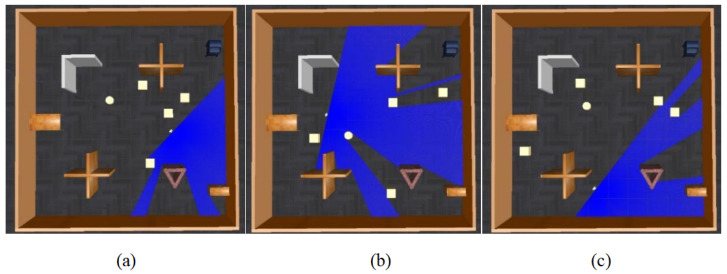
Example of the training environment in the gazebo simulation software. The blue lines are the laser beams. The cylinder acts as a moving obstacle that moves vertically, and the four boxes are obstacles that change randomly in each episode.

To enable the robot to autonomously avoid obstacles in real-world environments, it is necessary to learn a generalized obstacle avoidance strategy from sensor data. To enhance the generalizability of the learning algorithm and diversify the exploration of strategies, Gaussian noise is introduced into the sensors and the execution of actions. Additionally, to simulate dynamic obstacles in the real world and improve the robot’s ability to handle complex scenarios, a moving cylindrical obstacle and four boxes that change randomly with each training episode are introduced into the simulation training environment. These designs are intended to mimic the dynamism and uncertainty of the real world, allowing the robot to learn more robust and adaptable navigation strategies. The robot model trained in the simulated environment is consistent with the actual robot, and the starting position and target point position of the robot are randomly changed at the beginning of each episode. Figure 6 is an example of an environment trained in the gazebo simulation software.

**Figure 7:**
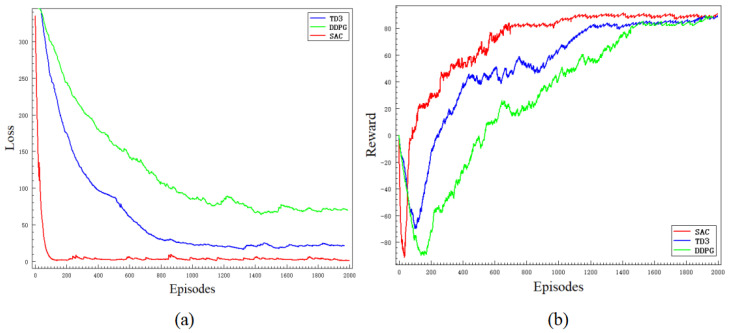
Training results of the deep reinforcement learning networks. The red line is the SAC algorithm, the blue line is the TD3 algorithm, and the green line is the DDPG algorithm.

Figure 7 depicts the convergence curves of loss and reward for the SAC, TD3, and DDPG algorithms under the conditions of sensor data fusion, reward function design, and the aforementioned environment during the training process. Each algorithm was trained for approximately 2000 episodes. As shown in Figure 7, the convergence speed of the SAC algorithm is notably faster than the other two algorithms, indicating that the SAC algorithm exhibits superior learning efficiency and performance. Due to the dynamic nature of the environment, significant fluctuations are observed in both the loss and reward curves.

### 4.3. Autonomous Exploration and Navigation

To quantitatively evaluate the performance of the proposed method in this study and accurately assess its effectiveness and efficiency in autonomous navigation tasks, we employed a comparative approach, contrasting it with various existing indoor navigation algorithms. Firstly, experiments were conducted using the SAC network without a global planner, referred to as the Local Deep Reinforcement Learning (L-DRL) method. Secondly, to compare the performance of the heuristic evaluation function, we compare the TD3+CPTD framework with the global navigation method [10] used in the heuristic navigation algorithm of [13], referring to them as NTD3 and OTD3, respectively. Considering that non-learning path planning algorithms struggle to achieve autonomous exploration and navigation in the absence of prior map information, experiments replaced the neural network in our proposed framework with the ROS local planner package, referred to as the LP method. Finally, to establish a performance benchmark for comparison, control experiments were conducted using the Dijkstra algorithm based on complete mapping. Each algorithm was tested in three different environments over five trials. Key recorded data included traveled distance (D, in meters), travel time (T, in seconds), and the number of successful goal arrivals (Arrive). Based on the collected experimental data, average traveled distance (Av.D) and average travel time (Av.T) were further calculated, along with maximum (Max.D, Max.T) and minimum (Min.D, Min.T) values for distance and time traveled. In this study, experiments were conducted in three different environments. To evaluate the transferability between simulation and real-world scenarios, Experiment 1 was performed in the Gazebo simulation software, while Experiments 2 and 3 were conducted in real-world environments.

**Figure 8:**
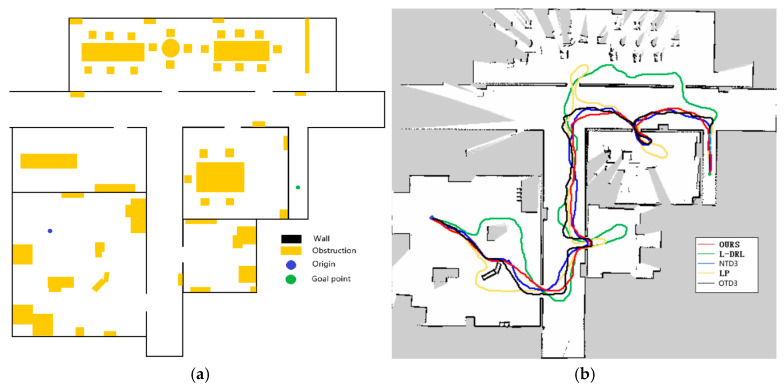
Environment and autonomous navigation path for Experiment 1. (**a**) is the description of the experimental environment, and (**b**) is an example of the autonomous navigation path.

The first experimental environment, as depicted in Figure 8, was designed with dense obstacles and multiple local optima regions. In this experiment, the method proposed in this study demonstrated efficient and precise navigation performance, successfully guiding the robot to the designated global target point. In contrast, the NTD3 algorithm exceeded the method proposed in this study in terms of travel time, although its travel path length was similar. Because the relationship between candidate point distances is taken into account, the path traveled by NTD3 is shorter than that of OTD3. The LP method, prone to becoming trapped in local optima and requiring a longer time to replan paths, resulted in longer travel distances. The L-DRL method exhibited looping behavior when navigating to local optima regions, especially in narrow gaps between obstacles, ultimately requiring human intervention to guide it out of such areas and into open spaces. Detailed experimental data are provided in Table 2.

**Figure 9:**
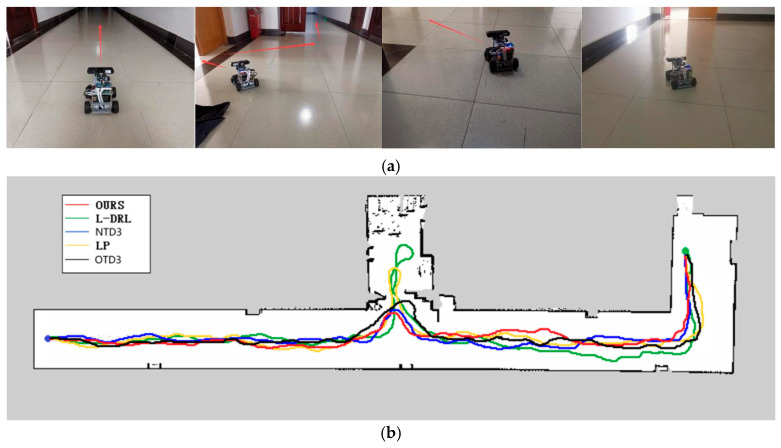
Environment and autonomous navigation path for Experiment 2. (**a**) is the description of the experimental environment, and (**b**) is an example of the autonomous navigation path.

The second experimental environment, as depicted in Figure 9, is primarily composed of a narrow corridor with smooth walls and contains few internal obstacles. The global target point coordinates are located at (33, −5). In this environment, each method was able to reach the global target point, but they exhibited differences in the length of the traveled path and the time required. The method proposed in this study not only rapidly reaches the global target point but also maintains the minimization of travel distance. Although the NTD3 algorithm is similar to our study’s method in terms of travel distance, its lower learning efficiency within the same training period compared to the SAC algorithm results in longer times required to execute certain actions. The path length traveled by OTD3 is still longer than that of NTD3. The LP method has a longer travel time due to the need to wait for the calculation of the next navigation point. The L-DRL method, on the other hand, is prone to falling into local optima, leading to a tendency to enter and delve into side paths. The specific experimental data can be found in Table 3.

**Figure 10:**
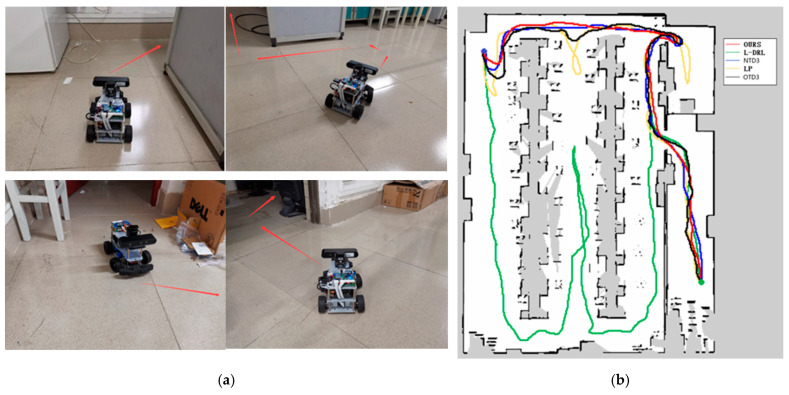
Environment and autonomous navigation path for Experiment 3. (**a**) is the description of the experimental environment, and (**b**) is an example of the autonomous navigation path.

The third experimental scenario, as illustrated in Figure 10, was conducted in a more complex environment featuring numerous obstacles such as desks, chairs, cardboard boxes, and keyboards. Particularly in the vicinity of keyboards, the feasible pathways are narrow, and failure to effectively recognize the keyboard may lead the robot to collide and become stuck, impeding its progress. The method proposed in this study can reach the designated global target point in the shortest time possible, with a relatively shorter travel path. Although the NTD3 algorithm is similar to our study’s method in terms of the length of the traveled path, its decision-making process takes longer. The performance of OTD3 is similar to that of NTD3, but its path is slightly longer, resulting in a longer time required. The LP method tends to become trapped in local optima, although it eventually breaks free, resulting in longer overall time and travel distance. Due to the lack of global planning capability, the L-DRL method tends to loop between the aisles of desks, struggling to break free from local optima, ultimately requiring human intervention to guide it into new areas. Detailed experimental data are provided in Table 4.

Synthesizing the experimental results, the method proposed in this study demonstrates consistent performance in both simulation and real-world environments. In simple environments as well as in complex environments with multiple local optima and numerous obstacles, it exhibits significant performance advantages over solutions based on the TD3 algorithm and planning-based methods. Although the TD3 algorithm showed similar performance to the method proposed in this study in some aspects during the experiments, the proposed method exhibited faster convergence and higher learning efficiency within the same training cycles. Compared to planning-based methods, the neural network-driven strategy can learn a wider range of motion patterns, enabling quicker escape from local optima. In contrast to the heuristic scoring method in [13], the CPTD method proposed in this study enables faster arrival at the global target, achieving quicker and more efficient global navigation.

## 5. Conclusions

In this study, we propose a robot autonomous navigation system based on deep reinforcement learning. Innovatively, this system employs the SAC algorithm as a local planner and combines it with a heuristic-based global planner to enhance the efficiency and accuracy of the robot’s global path planning. For obstacle detection and recognition, this research presents a strategy that fuses lidar sensor data with camera data, improving the robot’s accuracy in identifying obstacles in complex environments and enabling it to effectively navigate through narrow spaces. Additionally, the neural network module introduced in this system allows the robot to perform autonomous navigation tasks without pre-planning or prior information. Meanwhile, embedding a global navigation strategy, it compensates to some extent for the shortcomings of neural networks in global planning. Experimental results demonstrate that the performance of this system approaches the optimal solution achievable by non-learning planning algorithms based on prior map information. Ultimately, we aim for this system to inspire further research into neural network-based autonomous navigation systems, thereby advancing mobile robot technology and autonomous driving technology.

## Figures and Tables

**Table 1:** Specific scores for each point in Figure 4b.

Candidate Point	Point 1	Point 2	Point 3	Point 4	Point 5
Score	62.20	28.36	40.41	27.32	18.07

**Table 2:** Detailed experimental data for Experiment 1.

	Min.D (m)	Max.D (m)	Av.D (m)	Min.T (s)	Max.T (s)	Av.T (s)	Arrive
**OURS**	53.47	98.77	74.26	83.41	163.16	120.03	5/5
**L-DRL**	79.82	147.13	110.57	175.83	338.96	249.54	3/5
**NTD3**	55.24	99.16	75.21	97.64	180.29	134.82	5/5
**OTD3**	59.63	102.53	81.49	107.42	188.03	149.51	5/5
**LP**	69.53	122.06	94.59	150.12	273.41	211.98	5/5
**Dijkstra**	48.34	49.13	48.66	72.07	75.25	73.75	5/5

**Table 3:** Detailed experimental data for Experiment 2.

	Min.D (m)	Max.D (m)	Av.D (m)	Min.T (s)	Max.T (s)	Av.T (s)	Arrive
**OURS**	44.57	78.32	59.44	66.82	127.35	94.14	5/5
**L-DRL**	58.62	93.13	71.74	137.93	227.15	171.63	5/5
**NTD3**	47.28	81.63	61.25	86.65	156.09	115.33	5/5
**OTD3**	49.17	83.33	63.25	91.04	160.23	119.23	5/5
**LP**	50.58	86.91	65.57	120.13	208.93	156.84	5/5
**Dijkstra**	41.53	42.67	41.88	61.07	65.64	62.51	5/5

**Table 4:** Detailed experimental data for Experiment 3.

	Min.D (m)	Max.D (m)	Av.D (m)	Min.T (s)	Max.T (s)	Av.T (s)	Arrive
**OURS**	36.14	65.67	44.93	60.26	112.42	78.52	5/5
**L-DRL**	87.08	103.71	97.33	215.70	269.29	230.58	3/5
**NTD3**	39.27	66.24	45.71	72.04	121.84	84.84	5/5
**OTD3**	41.81	68.95	51.02	77.35	127.58	95.48	5/5
**LP**	49.34	90.59	64.56	115.51	210.92	152.46	5/5
**Dijkstra**	31.30	34.64	32.16	47.96	54.24	50.76	5/5

## Data Availability

No data were used for the research described in the article.
