# Correction: Ou et al. Autonomous Navigation by Mobile Robot with Sensor Fusion Based on Deep Reinforcement Learning. *Sensors* 2024, *24*, 3895

**DOI:** 10.3390/s25092780

**Published:** 2025-04-28

**Authors:** Yang Ou, Yiyi Cai, Youming Sun, Tuanfa Qin

**Affiliations:** 1 School of Computer and Electronic Information, Guangxi University, Nanning 530004, China; ouyang@st.gxu.edu.cn (Y.O.); caiyiyi@gxu.edu.cn (Y.C.); ymsun@gxu.edu.cn (Y.S.); 2 The Guangxi Key Laboratory of Multimedia Communications and Network Technology, Guangxi University, Nanning 530004, China; 3 School of Electronic and Information Engineering, South China University of Technology, Guangzhou 510641, China

## Text Correction

There were errors in the original publication [1].

1. In Paragraph 3 of Section 1, the item 2 has been updated as follows:

- Proposed a Candidate Point-Target Distance (CPTD) method, an improved heuristic algorithm that integrates a heuristic evaluation function into the navigation system of a four-wheeled mobile robot. This function is used to assess navigation path points and guide the robot toward the global target point.

2. In Section 3.2. Global Navigation, the parts of Paragraph 3 before Equation (6) have been updated as follows:

In the absence of sufficient environmental information, heuristic methods can effectively approximate the optimal solution to a problem. Based on this premise, the CPTD algorithm proposed in our study improves the scoring mechanism in [13], which evaluates all candidate navigation points through a heuristic function to select an optimal navigation point. The robot progressively moves towards each selected navigation point until reaching the global target point. At each step, the robot obtains its own position coordinates, coordinates of each candidate navigation point, coordinates of the global target point, and map data, enabling the calculation of the distances from the robot to each candidate navigation point and the global target point. Subsequently, the score of each candidate navigation point is computed based on the integrated map data. To enable the robot to reach the global target more quickly, we incorporate the previously selected candidate point as an evaluation factor in the function, ensuring that the robot will not choose a candidate point that is farther from the target under normal conditions. Given this information, the score, h of the *i*-th candidate navigation point, $c_{i}$, is determined as follows:

(5) $$h_{i}=S_{i,t}+\frac{1}{2}[D\left(c_{i},u\right)+D\left(m_{t},u\right)]+M_{i,t}$$

where $S_{i,t}$ is the distance score obtained based on the current position of the robot and the candidate navigation point, calculated as shown in Equation (6). $D\left(c_{i},u\right)$ is the Euclidean distance from the candidate navigation point to the global target point, calculated as shown in Equation (7). $D\left(m_{t},u\right)$ is the Euclidean distance from the previously selected candidate point to the global target point, calculated as shown in Equation (8). $M_{i,t}$ is the map information surrounding the candidate navigation point.

3. In Section 3.2. Global Navigation, the parts of Paragraph 4 before Equation (9) have been updated as follows:

Since there is no prior mapping, the robot can only acquire environmental information in real-time through its sensors. With each action, the robot’s map data are continuously refined in real-time. When a candidate navigation point is located within a known environment, the amount of information gained upon reaching that point is significantly less than that of a point situated in an unknown environment. Therefore, we prefer to guide the robot towards navigation points in unknown environments, which aids in discovering potential paths to the global target. Consequently, it is necessary to heuristically score each candidate navigation point based on the map data obtained. The status of every pixel in the map is identified, with unknown pixels marked as $p_{u}$, obstacles as $p_{o}$, and known pixels with no obstacles marked as $p_{f}$. To better reflect the score differences between known environments, unknown environments, and obstacles, we set the divisor to *k* to calculate the map information around the candidate point. The information score, M, for the environment within a [*k* × *k*] window around the candidate navigation point, ${\;c}_{i\;}$, is calculated as follows:

4. In Section 4.3. Autonomous Exploration and Navigation, the first paragraph has been updated as follows:

To quantitatively evaluate the performance of the proposed method in this study and accurately assess its effectiveness and efficiency in autonomous navigation tasks, we employed a comparative approach, contrasting it with various existing indoor navigation algorithms. Firstly, experiments were conducted using the SAC network without a global planner, referred to as the Local Deep Reinforcement Learning (L-DRL) method. Secondly, to compare the performance of the heuristic evaluation function, we compare the TD3+CPTD framework with the global navigation method [10] used in the heuristic navigation algorithm of [13], referring to them as NTD3 and OTD3, respectively. Considering that non-learning path planning algorithms struggle to achieve autonomous exploration and navigation in the absence of prior map information, experiments replaced the neural network in our proposed framework with the ROS local planner package, referred to as the LP method. Finally, to establish a performance benchmark for comparison, control experiments were conducted using the Dijkstra algorithm based on complete mapping. Each algorithm was tested in three different environments over five trials. Key recorded data included traveled distance (D, in meters), travel time (T, in seconds), and the number of successful goal arrivals (Arrive). Based on the collected experimental data, average traveled distance (Av.D) and average travel time (Av.T) were further calculated, along with maximum (Max.D, Max.T) and minimum (Min.D, Min.T) values for distance and time traveled. In this study, experiments were conducted in three different environments. To evaluate the transferability between simulation and real-world scenarios, Experiment 1 was performed in the Gazebo simulation software, while Experiments 2 and 3 were conducted in real-world environments.

5. In Section 4.3. Autonomous Exploration and Navigation, the second paragraph has been updated as follows:

The first experimental environment, as depicted in Figure 8, was designed with dense obstacles and multiple local optima regions. In this experiment, the method proposed in this study demonstrated efficient and precise navigation performance, successfully guiding the robot to the designated global target point. In contrast, the NTD3 algorithm exceeded the method proposed in this study in terms of travel time, although its travel path length was similar. Because the relationship between candidate point distances is taken into account, the path traveled by NTD3 is shorter than that of OTD3. The LP method, prone to becoming trapped in local optima and requiring a longer time to replan paths, resulted in longer travel distances. The L-DRL method exhibited looping behavior when navigating to local optima regions, especially in narrow gaps between obstacles, ultimately requiring human intervention to guide it out of such areas and into open spaces. Detailed experimental data are provided in Table 2.

6. In Section 4.3. Autonomous Exploration and Navigation, the third paragraph has been updated as follows:

The second experimental environment, as depicted in Figure 9, is primarily composed of a narrow corridor with smooth walls and contains few internal obstacles. The global target point coordinates are located at (33, −5). In this environment, each method was able to reach the global target point, but they exhibited differences in the length of the traveled path and the time required. The method proposed in this study not only rapidly reaches the global target point but also maintains the minimization of travel distance. Although the NTD3 algorithm is similar to our study’s method in terms of travel distance, its lower learning efficiency within the same training period compared to the SAC algorithm results in longer times required to execute certain actions. The path length traveled by OTD3 is still longer than that of NTD3. The LP method has a longer travel time due to the need to wait for the calculation of the next navigation point. The L-DRL method, on the other hand, is prone to falling into local optima, leading to a tendency to enter and delve into side paths. The specific experimental data can be found in Table 3.

7. In Section 4.3. Autonomous Exploration and Navigation, the fourth paragraph has been updated as follows:

The third experimental scenario, as illustrated in Figure 10, was conducted in a more complex environment featuring numerous obstacles such as desks, chairs, cardboard boxes, and keyboards. Particularly in the vicinity of keyboards, the feasible pathways are narrow, and failure to effectively recognize the keyboard may lead the robot to collide and become stuck, impeding its progress. The method proposed in this study can reach the designated global target point in the shortest time possible, with a relatively shorter travel path. Although the NTD3 algorithm is similar to our study’s method in terms of the length of the traveled path, its decision-making process takes longer. The performance of OTD3 is similar to that of NTD3, but its path is slightly longer, resulting in a longer time required. The LP method tends to become trapped in local optima, although it eventually breaks free, resulting in longer overall time and travel distance. Due to the lack of global planning capability, the L-DRL method tends to loop between the aisles of desks, struggling to break free from local optima, ultimately requiring human intervention to guide it into new areas. Detailed experimental data are provided in Table 4.

8. In Section 4.3. Autonomous Exploration and Navigation, the fifth paragraph has been updated as follows:

Synthesizing the experimental results, the method proposed in this study demonstrates consistent performance in both simulation and real-world environments. In simple environments as well as in complex environments with multiple local optima and numerous obstacles, it exhibits significant performance advantages over solutions based on the TD3 algorithm and planning-based methods. Although the TD3 algorithm showed similar performance to the method proposed in this study in some aspects during the experiments, the proposed method exhibited faster convergence and higher learning efficiency within the same training cycles. Compared to planning-based methods, the neural network-driven strategy can learn a wider range of motion patterns, enabling quicker escape from local optima. In contrast to the heuristic scoring method in [13], the CPTD method proposed in this study enables faster arrival at the global target, achieving quicker and more efficient global navigation.

## Figure Correction

1. The Caption of Figure 4 has been updated from “Figure 4. Heuristic function scoring example. The x and y coordinates are represented in meters. In (**a**), the z-coordinate represents the identification score of the pixels. The z-coordinate in (**b**,**c**) represents the score given by the heuristic function. (**a**) visualizes the environmental information. (**b**) displays the scores for four points, with the green point being the target point and the robot positioned at the origin (0,0). (**c**) shows the overall scores” to “Figure 4. Scoring example of the CPTD algorithm. The x and y coordinates are represented in meters. In (**a**), the z-coordinate represents the identification score of the pixels. The z-coordinate in (**b**,**c**) represents the score given by the heuristic function. (**a**) visualizes the environmental information. (**b**) displays the scores for four points, with the green point being the target point and the robot positioned at the origin (0,0). (**c**) shows the overall scores”.

2. The correct Figure 8, Figure 9 and Figure 10 has been updated as follows:

## Table Correction

The correct Table 2, Table 3 and Table 4 has been updated as follows:

The authors state that the scientific conclusions are unaffected. This correction was approved by the Academic Editor. The original publication has also been updated.

## Figures and Tables

**Figure 8:**
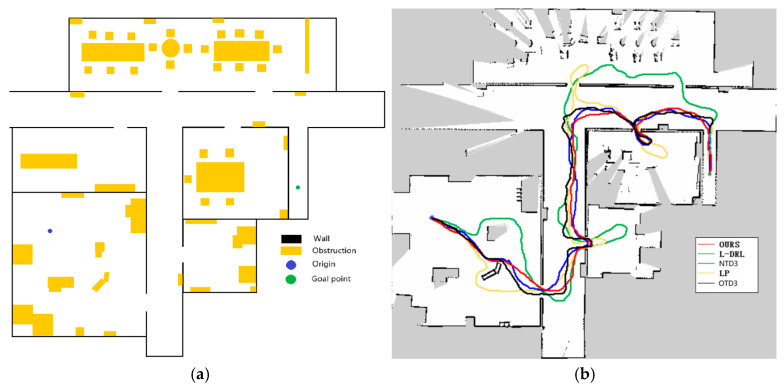
Environment and autonomous navigation path for Experiment 1. (**a**) is the description of the experimental environment, and (**b**) is an example of the autonomous navigation path.

**Figure 9:**
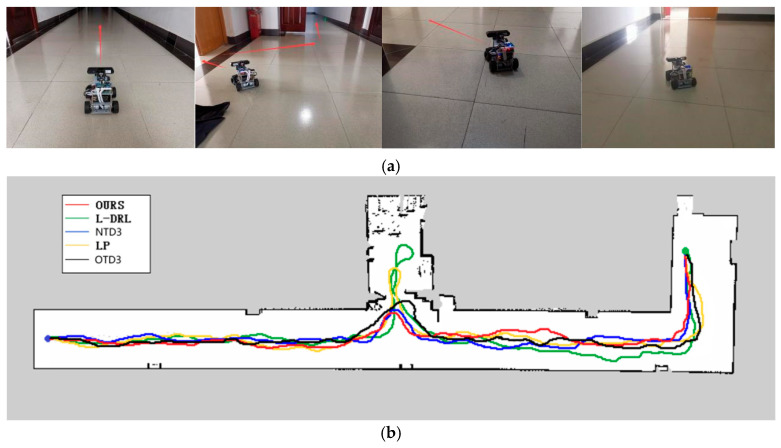
Environment and autonomous navigation path for Experiment 2. (**a**) is the description of the experimental environment, and (**b**) is an example of the autonomous navigation path.

**Figure 10:**
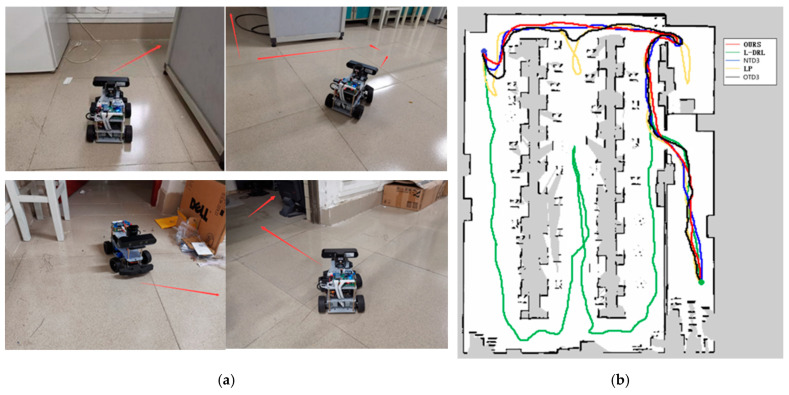
Environment and autonomous navigation path for Experiment 3. (**a**) is the description of the experimental environment, and (**b**) is an example of the autonomous navigation path.

**Table 2:** Detailed experimental data for Experiment 1.

	Min.D (m)	Max.D (m)	Av.D (m)	Min.T (s)	Max.T (s)	Av.T (s)	Arrive
**OURS**	53.47	98.77	74.26	83.41	163.16	120.03	5/5
**L-DRL**	79.82	147.13	110.57	175.83	338.96	249.54	3/5
**NTD3**	55.24	99.16	75.21	97.64	180.29	134.82	5/5
**OTD3**	59.63	102.53	81.49	107.42	188.03	149.51	5/5
**LP**	69.53	122.06	94.59	150.12	273.41	211.98	5/5
**Dijkstra**	48.34	49.13	48.66	72.07	75.25	73.75	5/5

**Table 3:** Detailed experimental data for Experiment 2.

	Min.D (m)	Max.D (m)	Av.D (m)	Min.T (s)	Max.T (s)	Av.T (s)	Arrive
**OURS**	44.57	78.32	59.44	66.82	127.35	94.14	5/5
**L-DRL**	58.62	93.13	71.74	137.93	227.15	171.63	5/5
**NTD3**	47.28	81.63	61.25	86.65	156.09	115.33	5/5
**OTD3**	49.17	83.33	63.25	91.04	160.23	119.23	5/5
**LP**	50.58	86.91	65.57	120.13	208.93	156.84	5/5
**Dijkstra**	41.53	42.67	41.88	61.07	65.64	62.51	5/5

**Table 4:** Detailed experimental data for Experiment 3.

	Min.D (m)	Max.D (m)	Av.D (m)	Min.T (s)	Max.T (s)	Av.T (s)	Arrive
**OURS**	36.14	65.67	44.93	60.26	112.42	78.52	5/5
**L-DRL**	87.08	103.71	97.33	215.70	269.29	230.58	3/5
**NTD3**	39.27	66.24	45.71	72.04	121.84	84.84	5/5
**OTD3**	41.81	68.95	51.02	77.35	127.58	95.48	5/5
**LP**	49.34	90.59	64.56	115.51	210.92	152.46	5/5
**Dijkstra**	31.30	34.64	32.16	47.96	54.24	50.76	5/5

## References

[1] Ou Y., Cai Y., Sun Y., Qin T. (2024). Autonomous Navigation by Mobile Robot with Sensor Fusion Based on Deep Reinforcement Learning. Sensors.

